# Disruption of normal brain distribution of [^18^F]Nifene to α4β2* nicotinic acetylcholinergic receptors in old B6129SF2/J mice and transgenic 3xTg-AD mice model of Alzheimer’s disease: *In Vivo* PET/CT imaging studies

**DOI:** 10.1016/j.neuroimage.2025.121092

**Published:** 2025-02-18

**Authors:** Christopher Liang, Atsumi A. Okamoto, Fariha Karim, Shimako Kawauchi, Lusine Melkonyan, Tram B. Danh, Jogeshwar Mukherjee

**Affiliations:** a Preclinical Imaging, Department of Radiological Sciences, University of California-Irvine, Irvine, CA, United States; b Transgenic Mouse Facility, University Laboratory Animal Resources, Office of Research, University of California-Irvine, Irvine, CA, United States

**Keywords:** [^18^F]Nifene, PET/CT, 3xTg-AD mice, B2169SF2/J mice, [^125^I]IBETA, Alzheimer’s disease

## Abstract

The 3xTg-AD transgenic mouse model develops Aβ plaque and tau pathology and is purported to closely resemble pathological development in the human Alzheimer’s disease (AD) brain. Nicotinic acetylcholine receptors (nAChRs) α4β2* subtype, was studied in this mouse model using [^18^F]nifene PET/CT and compared with non-transgenic B6129SF2/J mice (male and female). Young 2-month old B6129SF2/J exhibited normal [^18^F]nifene distribution (measured as standard uptake volume ratios, SUVR with cerebellum as reference) thalamus (TH) 3.12> medial prefrontal cortex (mPFC) 2.33> frontal cortex (FC) 2.06> hippocampus-subiculum (HP-SUB) 1.6. At 11-months of age, B6129SF2/J exhibited high, irreversible and non-saturable [^18^F]nifene binding in mPFC higher than in TH (mPFC 3.8> TH 2.82> FC 1.79> HP-SUB 1.73). The 3xTg-AD also exhibited high mPFC binding, although the region of highest binding within the mPFC was different compared to B6129SF2/J mice (mPFC 2.44> TH 2.27> FC 1.61> HP-SUB 1.48). [^125^I]IBETA and immunohistochemistry in 3xTg-AD brain slices confirmed Aβ plaques. The TH of 3xTg-AD mice had lower [^18^F]nifene binding (reduced by approximately 20 %) compared to both, young and old B6129SF2/J, and was significant. The mPFC [^18^F]nifene binding was significantly higher in the old B6129SF2/J compared to both the young B6129SF2/J and the 3xTg-AD mice (>150 %). Overall, 3xTg-AD transgenic mice had reduced [^18^F]nifene binding compared to B6129SF2/J controls, suggesting possible effects of Aβ plaques and Tau on α4β2* nAChRs.

## 1. Introduction

Cholinergic pathway, including the α4β2* nicotinic acetylcholine receptor (nAChR) subtype in the brain, is involved in cognitive functions such as attention, learning and memory (Bieszczbad et al., 2012). Deficiency of α4β2* nAChRs constitute a major subtype lost in human Alzheimer’s disease (AD) brains (Sultzer et al., 2022; Sabri et al., 2018). Because of this deficiency, treatment with acetylcholinergic inhibitors (AChEI) has been found beneficial in the treatment of early onset AD (Zuin et al., 2022; Marucci et al., 2021). Transgenic mice models allow the study of specific pathologies such as Aβ plaques and neurofibrillary tangles (NFT, tau) in AD (Webster et al., 2014). We recently reported PET/CT studies using [^18^F]nifene, an α4β2* nAChR probe in the 5xFAD transgenic mice (Liang et al., 2023). Our findings using brain slices in vitro autoradiography and in vivo PET confirmed Aβ plaque accumulation in 5xFAD mice using the imaging agent [^124/125^I]IBETA and [^18^F] flotaza (Nguyen et al., 2022; Sandhu et al., 2024). A major finding is the anomalous higher, non-displaceable binding of [^18^F]nifene in the medial prefrontal cortex (mPFC). It was unclear if [^18^F]nifene binding in mPFC suggested a compensatory mechanism against Aβ pathology. Nicotine was able to displace [^18^F]nifene binding in the thalamus (TH) but the irreversibility and inability of nicotine to displace in vivo [^18^F]nifene in mPFC of 5xFAD mice was surprising. There did not appear to be an overlap between high levels of Aβ plaques and [^18^F]nifene in the mPFC, although adjacent regions of anterior cingulate cortex (ACC) had similar levels of the two biomarkers. The higher [^18^F]nifene binding in the mPFC could be due to entrapment by microglia, which are known to be present in the 5xFAD mice (Crapser et al., 2020).

The attractive combination of both Aβ plaque and tangle development makes the 3xTg-AD mouse regarded as a more complete transgenic mouse model of AD pathology to study the effects on α4β2* nAChR. The 3xTg-AD transgenic mice express Aβ plaques and Tau and may reflect human AD by including both pathologies (Javonillo et al., 2022). The 3xTg-AD model develops age-related and progressive amyloid and tau pathologies, with extracellular plaques appearing at 6–12 months of age, followed by NFTs becoming apparent at 12-months of age. Additionally, 3xTg-AD mice have also displayed localized neurodegeneration, synaptic impairment, and cognitive deficits at 6 months of age. Using the B6129SF2/J strain as a control wild-type species, the 3xTg-AD mice were developed (Javonillo et al., 2022). A number of comparative behavior, pathology and pharmacological studies have been carried out on the 3xTg-AD mice using B6129SF2/J as the controls. Studies have shown distinct differences between the 3xTg-AD and B6129SF2/J mice in learning and memory tasks (Russo-Savage et al., 2020). There have been few reports on the development of microglia in the B6129SF2/J mice and the potential changes in triggering receptor expressed on myeloid cells (TREM1 and TREM2) expression with age (Javonillo et al., 2022; Ochi et al., 2020). None of the reported studies on B6129SF2/J mice revealed any significant deviation from a normal wild type mice behavior. Thus, B6129SF2/J mice would serve as a good control group for comparative imaging study of α4β2* nAChR in the 3xTg-AD mice.

**Fig. 1.**
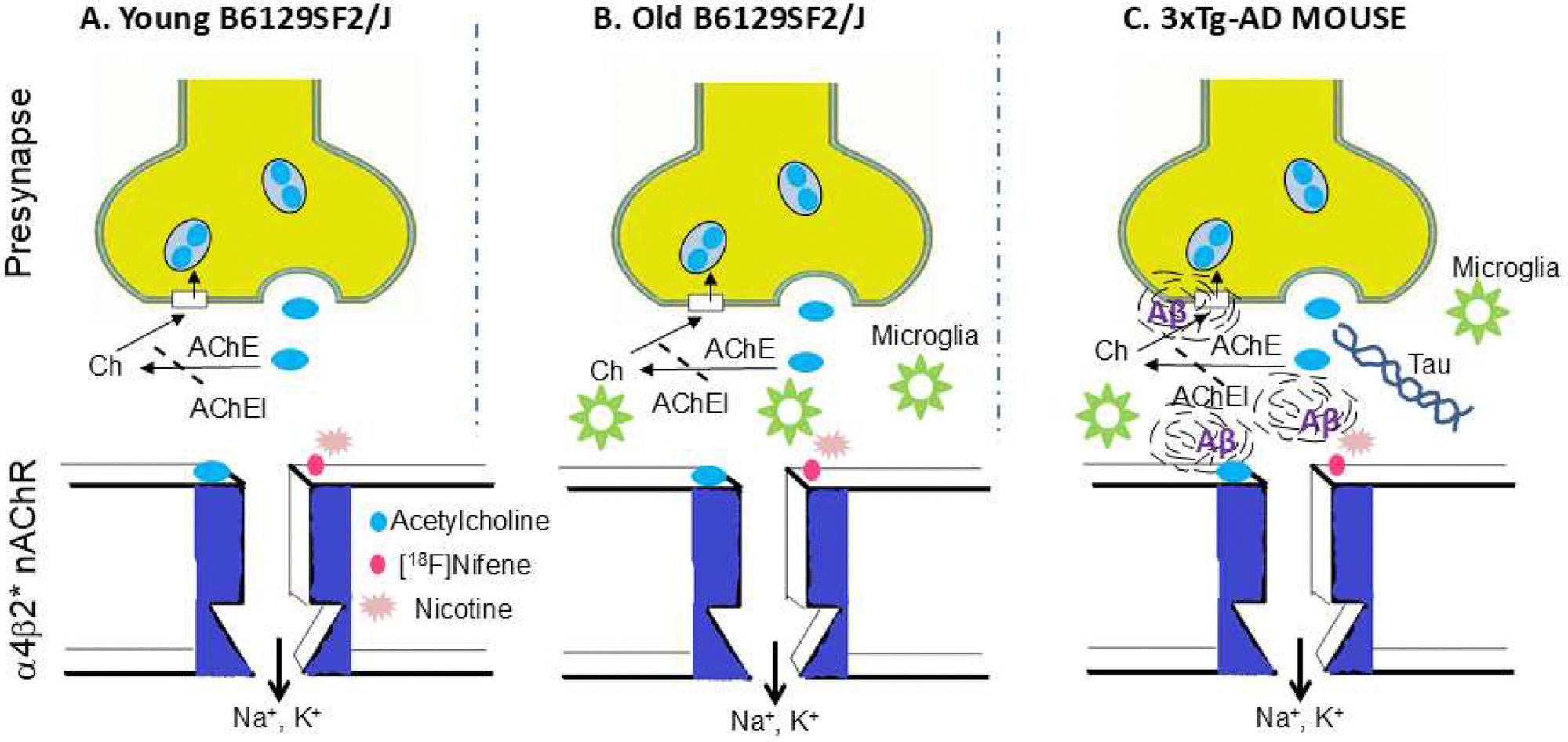
Schematic of a4b2* nAChRs in 3xTg-AD mice: (A). Schematic showing synaptic junction of α4β2* nAChR in young (2 month) B6129SF2/J normal mouse; (B). In older (11 month) B6129SF2/J mouse, microglia present in the synapses may interact and/or interrupt [^18^F]nifene, acetylcholine and nicotine binding; (C). In 3xTg-AD transgenic mouse, Aβ plaques, Tau and microglia present in the synapses may interact and/or interrupt [^18^F]nifene, acetylcholine and nicotine binding (nAChR: nicotinic acetylcholine receptor; AChE: acetylcholinesterase; Ch: choline; AChEI: acetylcholinesterase inhibitors).

Schematic in Fig. 1 shows the three groups of mice that were investigated: Group #1, B6129SF2/J mice, male and female starting at age 2 months; Group #2, B6129SF2/J mice, male and female starting at age 11 months, as a control group for 3xTg mice; Group #3, 3xTg-AD mice, male and female starting at age 11 months. Uninterrupted normal interaction of nicotine and [^18^F]nifene are expected in the young B6129SF2/J (Fig. 1A). Although older B6129SF2/J mice are expected to be similar to the younger mice, aging effects have suggested increased levels of microglia in the mice (Fig. 1B; Javonillo et al., 2022). The 3xTg-AD mice expressing Aβ plaques and NFT tau as shown in Fig. 1C, age and sex matched with older B6129SF2/J mice was examined potential alterations in α4β2* nAChRs using in vivo PET/CT studies. The three groups of mice in Fig. 1 were investigated using in vivo PET/CT [^18^F]nifene imaging studies and nicotine challenge studies to evaluate reversibility of [^18^F]nifene binding. Ex vivo autoradiographic studies were used to further assess the nature of [^18^F]nifene binding in the mice. In vitro Aβ plaques autoradiography was carried out using [^125^I]IBETA (Nguyen et al., 2022; Mondal et al., 2023). Findings from this study will have translational potential of [^18^F]nifene in human AD studies (Mukherjee et al., 2018).

## 2. Materials and methods

### 2.1. General methods

Fluorine-18 fluoride in oxygen-18 enriched water was purchased from PETNET, Inc (Culver City, CA). Iodine-125 was purchased from American Radiolabeled Chemicals, Inc (St Louis, MO). Fluorine-18 and iodine-125 radioactivity were counted in a Capintec CRC-15R dose calibrator. All solvents used were provided by Fisher Scientific. Gilson high performance liquid chromatography (HPLC) was used for the semi-preparative reverse-phase column (10 × 250 mm 10 micron Econosil C18 reverse-phase) chromatography with UV detector set at dual wavelengths of 254 nm and 280 nm as well as a radioactivity detector. RadioTLC were scanned on an AR-2000 imaging scanner (Eckart & Ziegler, Berlin, Germany). Mice brain slices were prepared at 10 μm to 40 μm thick using the Lieca 1850 cryotome. In vitro or ex vivo labeled brain sections were exposed to phosphor films (Perkin Elmer Multi-sensitive, Medium MS) and read using the Cyclone Phosphor Imaging System (Packard Instruments). Analysis of in vitro or ex vivo autoradiographs was done using Optiquant acquisition and analysis software.

### 2.2. Radiopharmaceuticals

The radiosynthesis of [^18^F]nifene using [^18^F]fluoride was previously described (Pichika et al., 2006; Campoy et al., 2021). The purified [^18^F]nifene was in sterile saline (0.9 % NaCl injection). Radiochemical purity of [^18^F]nifene was >98 % and chemical purity was found to be >95 % with a measured molar activity >70 GBq/μmol (>2 Ci/μmol) at the end of synthesis. [^125^I]IBETA known for selective binding to both 5xFAD mice and human AD brain Aβ plaques was used for autoradiography of 3xTg-AD and B6129SF2/J mice brain slices (Nguyen et al., 2022; Sandhu et al., 2024). Radiochemical purity of [^125^I]IBETA was >95 % with molar activity >500 GBq/μmol (>13 Ci/μmol).

### 2.3. Animals

All animal studies were approved by the Institutional Animal Health Care and Use Committee of University of California-Irvine. Transgenic 3xTg-AD mice, homozygous for presenilin1 (Psen1) mutation, homozygous for APPSwe and tauP301 L transgenes (B6;129-Tg(APPSwe, tauP301 L) 1Lfa Psen1^tm1Mpm^/Mmjax, MMRRC stock number 034,830-JAX) (female, *n* = 4 and male, *n* = 4, age 11 months, and their age and gender controls (B6129SF2/J, female, *n* = 4 and male, *n* = 4, age 11 months) were obtained from UCI MODEL-AD consortium (Forner et al., 2021). The F2 hybrids are used as controls for genetically engineered 3xTg-AD strains that were generated with 129-derived embryonic stem cells and maintained on a mixed B6129 background. Younger B6129SF2/J mice (JAX stock #101,045), female, *n* = 4 and male, *n* = 4, age 2 months were purchased from Jackson Laboratory (Table 1). Mice were housed under controlled temperatures of 22 °C ± 1 °C, in a 12-h light–dark cycle, on at 6:00AM, with water and food chow ad libitum. All animals recovered from the isoflurane anesthesia after the PET/CT imaging procedures.

### 2.4. PET/CT imaging 3xTg-AD mice [^18^F]Nifene

The Inveon PET and MM CT scanners were placed in the “docked mode” for combined PET/CT experiments. All PET images were calibrated in units of Bq/cm^3^ by scanning a Ge-68 cylinder (6 cm diameter) with known activity and reconstructing the acquired image with parameters identical to those of [^18^F]nifene images (Constantinescu and Mukherjee, 2009). The CT images were spatially transformed to match the reconstructed PET images (Liang et al., 2023).

Male and female transgenic 3xTg-AD mice and control B6129SF2/J mice male and female were used in the study (Table 1). All mice were fasted for at least 18 h prior to the imaging study. All mice were injected [^18^F]nifene intraperitoneally in sterile saline under 2 % isoflurane (St. Joseph, MO). For static scans, mice were awake after [^18^F]nifene injections and free to move in their cages. For dynamic scans, injected mice were placed in the supine position in a mouse holder and anesthetized with 2 % isoflurane for whole-body PET/CT imaging. Dynamic PET scans were acquired for 2 h after [^18^F]nifene injections (Constantinescu et al., 2013). Before the PET scan, the scanner bed moved to the CT for a 7.5-minute-long CT scan which was used for attenuation correction and anatomical delineation of PET images. The Inveon multimodality scanner was used for all combined PET/CT experiments. For static scans, injected mice were placed in the supine position in a mouse holder 40 min after [^18^F]nifene injections and anesthetized with 2 % isoflurane. A 7.5-minute-long CT scan for attenuation correction and anatomical delineation of PET images was followed by a 30 min-long PET scan. The Inveon multimodality scanner was used for all combined PET/CT experiments.

### 2.5. 3xTg-AD mice [^18^F]Nifene ex vivo autoradiography

After [^18^F]nifene in vivo PET/CT experiments in B6129SF2/J and 3xTg-AD mice, select mice were killed and the brain was excised. The brains containing [^18^F]nifene were rapidly frozen at −80 °C for approximately 10 min, followed by 10 min in the Leica cryotome at −20 °C. Horizontal brain sections (40μm thick) containing TH, subiculum (SUB), cortex, striatum, hippocampus (HP), and cerebellum (CB) were cut using the Leica cryotome. The sections were rapidly air dried and subsequently apposed to phosphor films overnight. Films were read using the Cyclone Phosphor Imaging System. Regions-of-interest were drawn and analyzed on brain regions rich in α4β2* nicotinic receptors using OptiQuant software and binding of [^18^F]nifene measured in Digital Light Units/mm^2^ (DLU/mm^2^).

### 2.6. In vivo nicotine effects 3xTg-AD mice [^18^F]Nifene PET/CT

To assess effects of nicotine treatment in vivo, B6129SF2/J and 3xTg-AD mice were used. Nicotine was administered either after [^18^F]nifene (post-nicotine) or before [^18^F]nifene (pre-nicotine) administration. All mice were fasted for at least 18 h prior to the imaging study. All mice were injected [^18^F]nifene (intraperitoneally in sterile saline) under 2 % isoflurane (St. Joseph, MO). They were placed in the supine position in a mouse holder and anesthetized with 2 % isoflurane for whole-body PET/CT imaging. A 30 min long dynamic PET scans was acquired after [^18^F]nifene injections followed by a 7.5-minute-long CT scan after the PET scan for attenuation correction. Nicotine (1 mg/kg, administered as a nicotine ditartarate salt in sterile saline, 0.1 mL, IP) was administered to the mice while in the scanner bed and a second 30-minute dynamic PET scan post-nicotine injection was acquired.

### 2.7. [^125^I]IBETA 3xTg-AD mice brain in vitro autoradiography for Aβ plaques

In order to assess Aβ plaque load in the 3xTg-AD mice brain slices, in vitro studies using [^125^I]IBETA were carried out using our previously described procedures (Nguyen et al., 2022). Mice brain slices taken from age-matched 3xTg-AD, 5xFAD and B6129SF2/J mice (10 μm thick) were treated with [^125^I]IBETA in 40 % ethanol PBS buffer pH 7.4 (60 mL; 1.5 kBq/mL) were incubated at 25 °C for 1.25 hr. The slices were then washed with cold PBS buffer, 90 % ethanolic PBS buffer twice, PBS buffer and cold water. The brain sections were air dried, exposed overnight on a phosphor film, and then read on the Cyclone Storage Phosphor System. The amount of bound [^125^I]IBETA in the autoradiograms was evaluated in various brain regions as digital lights units (DLU/mm^2^) using the OptiQuant program (Packard Instruments Co.).

### 2.8. Immunohistochemistry

Immunohistochemical (IHC) staining of all brain sections were carried out by University of California-Irvine, Pathology services using Ventana BenchMark Ultra protocols. To determine the localization of Aβ accumulation, neighboring mouse brain slices (10 μm thick) from in vitro were immunostained with anti-Aβ Biolegend 803,015 (Biolegend, CA, USA). Immunostained sections were scanned using the Ventana Roche instrumentation and the images were analyzed using QuPath software (Mondal et al., 2023).

### 2.9. Image analysis

Inveon Research Workplace (IRW) software (Siemens Medical Solutions, Knoxville, TN) and PMOD Software (PMOD Technologies, Zurich, Switzerland) were used to analyze all in vivo PET/CT images (Liang et al., 2023). Whole-body PET/CT images were analyzed using the IRW software for [^18^F]nifene uptake. Using PMOD, PET images were co-registered to a mouse brain MRI template (Mondal et al., 2021; Campoy et al., 2021; Liang et al., 2023). The magnitude of [^18^F]nifene was expressed as standard uptake value (SUV) which was computed as the average [^18^F]nifene activity in each volume of interest, VOI (in kBq/mL) divided by the injected dose (in MBq) times the body weight of each animal (in Kg). Regional values were obtained from 30-minute static scans taken 40 mins after injection of [^18^F]nifene. Using CB SUV as the reference region, SUVR (standard uptake value ratio) was calculated to compare different groups of mice. The SUV and SUVR values were then statistically analyzed using GraphPad Prism 9 and 3xTg-AD mice were compared with B6129SF2/J mice.

## 3. Results

### 3.1. Young B6129SF2/J [^18^F]Nifene PET/CT

Uptake of [^18^F]nifene was observed in various brain regions of the 2-month old young B6129SF2/J mouse brain was similar to our previous findings with the BALB/c mice and C57BL/J mice (Campoy et al., 2021; Liang et al., 2023; Zhang et al., 2023). Normal [^18^F]nifene distribution in the mouse brain shows highest uptake in the TH followed by frontal cortex (FC), which included ACC in the mPFC region and SUB region near the hippocampus (HP-SUB) and the least binding in the CB. Cerebellum is therefore used as a reference region in the mice where it is assumed that there are the least number of α4β2* nAChRs. Harderian glands exhibited high nonspecific activity as previously reported in other mice species (Campoy et al., 2021).

**Fig. 2.**
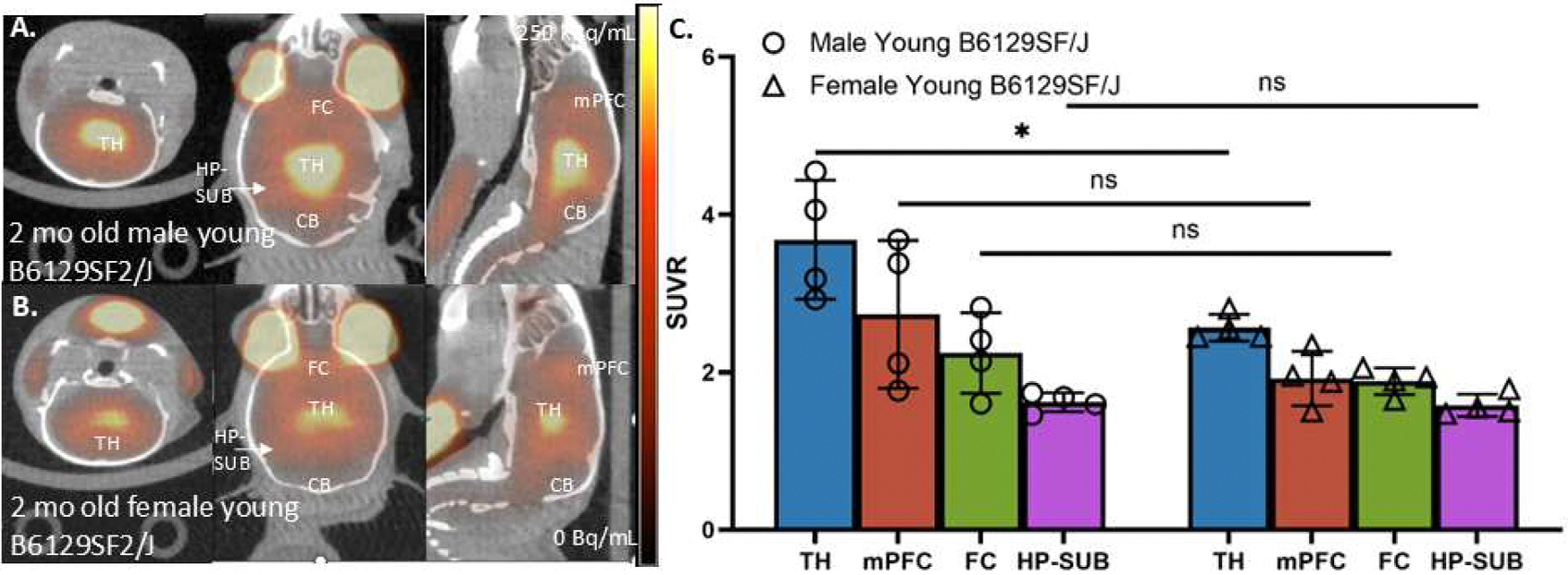
Young (2 month old) B6129SF/J mice [^18^F]Nifene PET/CT: (A). [^18^F]Nifene in B6129SF/J mice (2 month male, 20*g*, 6.29 MBq, IP) showing binding in TH, FC, mPFC and low binding in CB with [^18^F]nifene greater in TH compared to other regions; (B). [^18^F]Nifene in B6129SF/J mice (2 month female, 18*g*, 4.95 MBq, IP) showing binding in TH, FC, mPFC and low binding in CB with [^18^F]nifene greater in TH compared to FC; (C). Bar graph comparing SUVR averages (CB as reference) of B6129SF2/J male (*n* = 4) and female (*n* = 4) in TH, mPFC and FC. Differences in [^18^F]nifene TH binding between the two groups was significant (*p* = 0.03) with males showing higher binding. Differences in other regions (mPFC, FC and HP-SUB) were not significant. (TH=thalamus; FC=frontal cortex; mPFC= medial prefrontal cortex; HP-SUB=hippocampus-subiculum; CB=cerebellum; HG=harderian gland).

Fig. 2A,B shows results of the static [^18^F]nifene PET/CT scans with the highest levels of [^18^F]nifene in TH in both male and female 2-month old B6129SF2/J mice. Using the CB of each mouse as its internal reference region, SUVR values were derived for all mice as shown in Fig. 2C. The rank order of the binding of [^18^F]nifene was TH> mPFC≥ FC> HP-SUB. Males had higher average levels in TH (SUVR=3.68) compared to the females (SUVR=2.56) and was found to be significant (*p* < 0.05) (Fig. 2C). Other brain regions (mPFC, FC, HP-SUB) exhibited normal order of distribution of [^18^F]nifene in both the male and female groups, consistent with our findings in other mice species (Liang et al., 2023; Campoy et al., 2021). The average SUVR values in the male young group were marginally higher (mPFC=2.73; FC=2.24; HP-SUB=1.62) compared to the female young mice (mPFC=1.92; FC=1.88; HP-SUB=1.58). However, they were not found to be statistically significant.

### 3.2. Old B6129SF2/J [^18^F]Nifene PET/CT

**Fig. 3.**
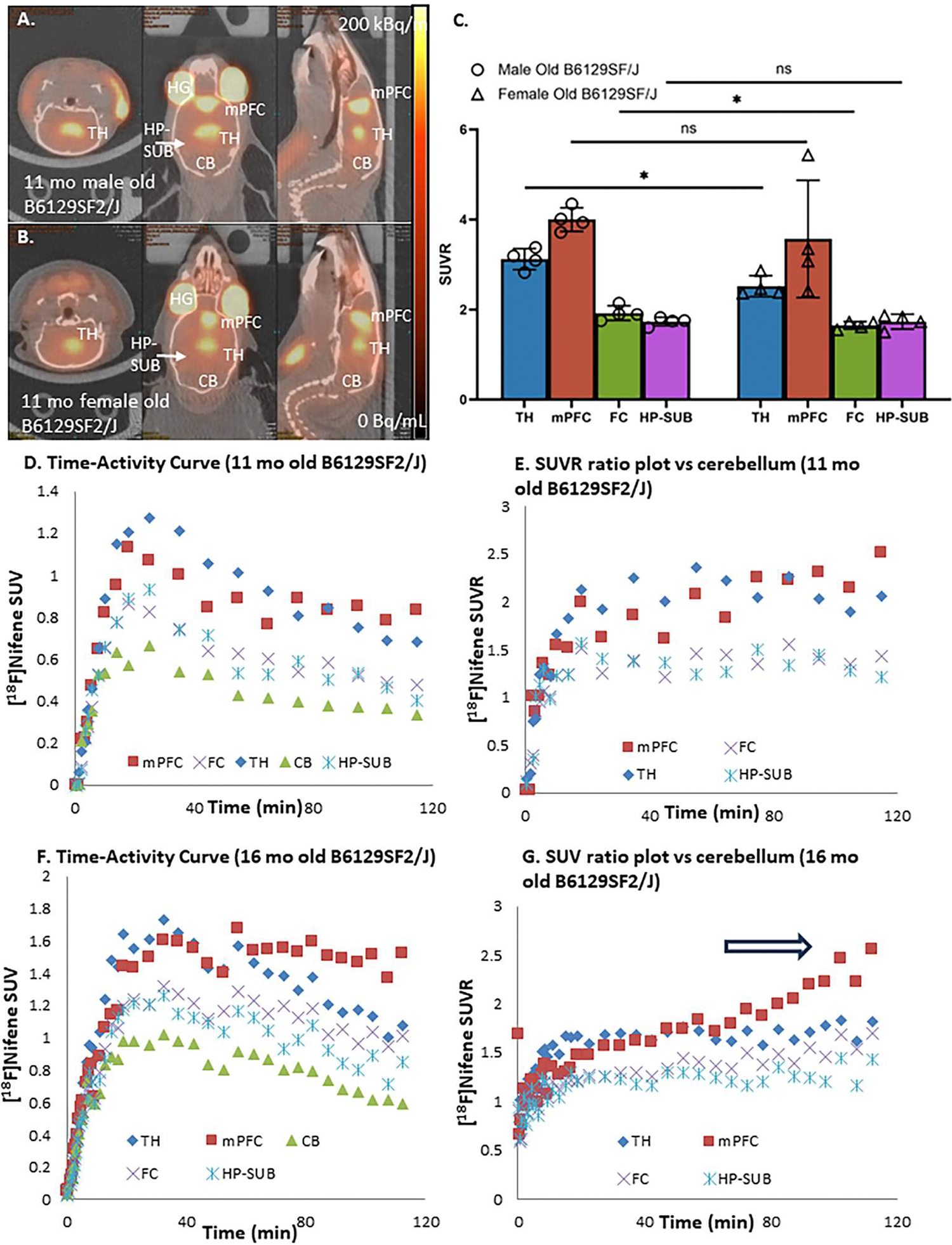
Older (11–16 month old) B6129SF/J mice [^18^F]Nifene PET/CT: (A). [^18^F]Nifene in B6129SF/J mice 11 month male, 30*g* , 6.62 MBq, IP) showing binding in TH, FC, mPFC, HP-SUB and low binding in CB with [^18^F]nifene greater in mPFC compared to other regions; (B). [^18^F]Nifene in B6129SF/J mice (11 month female, 32*g* , 5.62 MBq, IP) showing binding in TH, FC, mPFC, HP-SUB and low binding in CB with [^18^F]nifene greater in mPFC compared to other regions; (C). Bar graph comparing SUVR averages (CB as reference) of B6129SF2/J male (*n* = 4) and female (*n* = 4) in TH, mPFC, FC and HP-SUB. Differences in [^18^F]nifene TH and FC binding between the two groups were significant (*p* = 0.01(TH) and *p* = 0.03(FC)) with males showing higher binding. Differences in mPFC and HP-SUB were not significant (TH=thalamus; FC=frontal cortex; mPFC=medial prefrontal cortex; HP-SUB= hippocampus-subiculum; CB=cerebellum; HG=harderian gland). (D). Time activity curve of [^18^F]nifene in 11-month old male B6129SF/J (64*g* , 21.42 MBq, IP) showing uptake and clearance from various brain regions, except mPFC which showed slower clearance. (E). Ratio plot versus CB (SUVR) shows a ~2.05 of TH, while mPFC remained over ~2.30 at the end of the scan; (F). Time activity curve of [^18^F]nifene in 16-month old male B6129SF/J (32*g*, 13.24 MBq, IP) showing uptake and clearance from various brain regions, except mPFC which showed retention of [^18^F]nifene; (G). Ratio plot versus CB (SUVR) shows a SUVR of 1.8 for TH, while mPFC continued to rise (arrow) over 2.6 at the end of the scan suggesting retention of [^18^F]nifene.

Older B6129SF2/J mice (11-month old from UCI Model-AD) were first used in the static [^18^F]nifene PET/CT scans. The accumulation of [^18^F]nifene in the brains of the older B6129SF2/J mice looked very different from the younger 2-month old B6129SF2/J mice (Fig. 3A,B). The highest levels of [^18^F]nifene binding was found in the mPFC, in both males and females, as opposed to the TH in the younger animals. Thus, the rank order of the binding of [^18^F]nifene changed to mPFC> TH> FC≥ HP-SUB. The higher mPFC binding of [^18^F]nifene was consistently seen in all old B6129SF2/J mice. The SUVR values of old male mice were: mPFC=3.99; TH=3.12; FC=1.92; HP-SUB=1.73 which were generally higher compared to the old female mice SUVR: mPFC=3.57; TH=2.52; FC=1.65; HP-SUB=1.73. Differences between old male and old female TH and FC were found to be statistically significant (*p* < 0.05) as shown in Fig. 3C. The higher SUVR values in male B61229SF2/J mice compared to the female B6129SF2/J mice was consistent in both the 2-month old young group as well as the 11-month older group. The major difference was the significantly higher mPFC [^18^F]nifene binding in the older B6129SF2/J male and female mice compared to the 2-month old mice.

Dynamic PET/CT [^18^F]nifene scans were carried out in the older B6129SF2/J mice in order to evaluate the time course of binding in the various brain regions. Time-activity curve of SUV in Fig-3D of 11-month old B6129SF2/J mouse shows progressive increase of [^18^F]nifene uptake in all brain regions (TH, mFPC, FC, HP-SUB and CB) peaking at approximately 30 mins post-intraperitoneal injection. Subsequently, [^18^F]nifene cleared from all brain regions, with the slowest clearance from mPFC. Using CB as the reference region, the SUVR ratio plot for the 11-month old B6129SF2/J mouse is shown in Fig. 3E. The ratio plot for TH, FC and HP-SUB is consistent with our reported findings in other mice species (Campoy et al., 2021). However, the ratio plot of mPFC was unusually higher, and displayed a trend to remain high and continue to increase. By the end of the scan, SUVR for mPFC was found to be higher compared to TH (SUVR TH=2.05, mPFC=2.30, FC=1.35, HP-SUB=1.21). This abnormal uptake in the mPFC was not found in our PET/CT imaging studies with other species (Campoy et al., 2021; Constantinescu et al., 2013), except the 5xFAD mice (Liang et al., 2023).

**Fig. 4.**
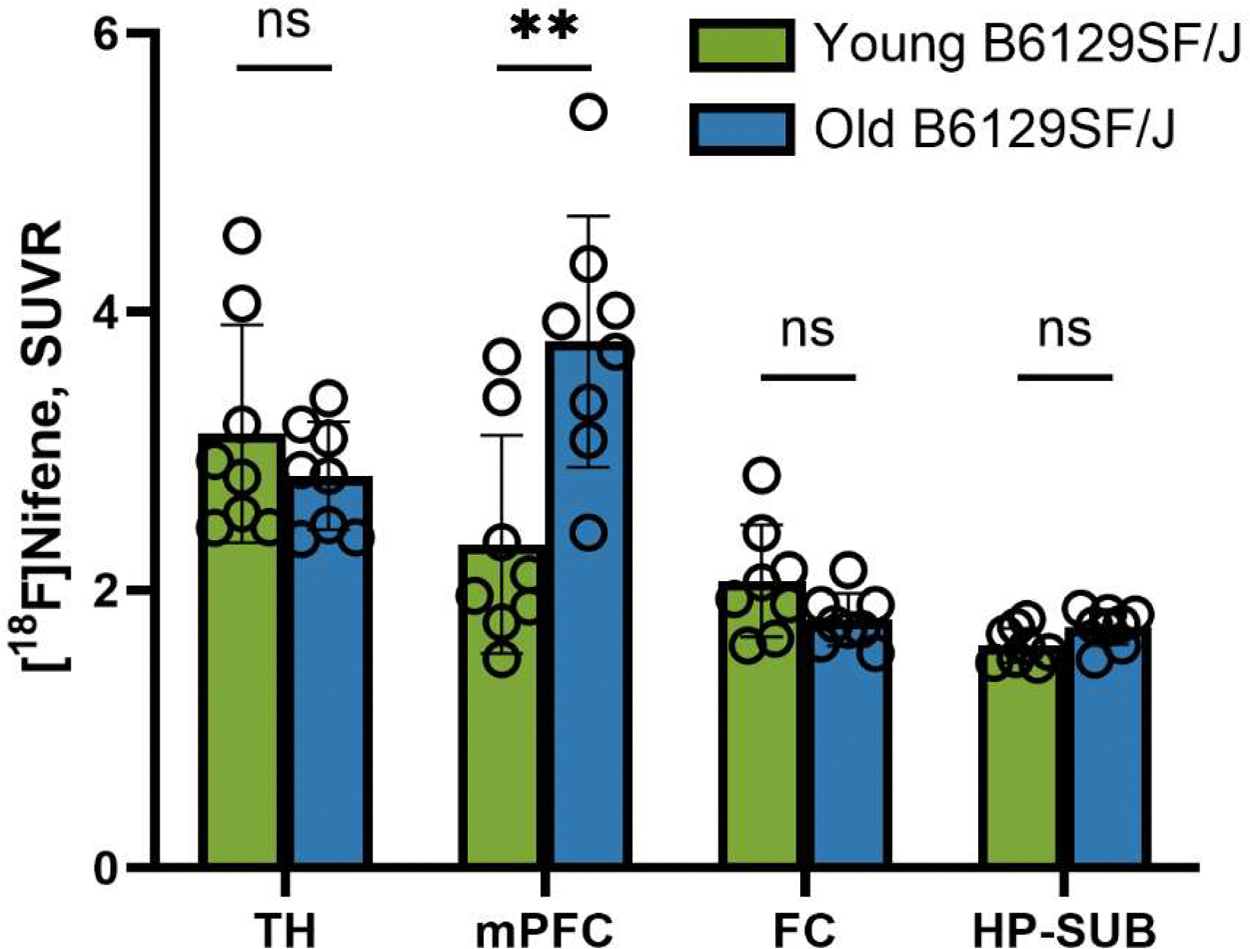
Comparison of [^18^F]Nifene PET/CT in young versus old B6129SF/J Mice: TH: Young B6129SF/J vs. Old B6129SF/*J* = 0.3428 (ns); mPFC: Young B6129SF/J vs. Old B6129SF/*J* = 0.0039 (**); FC: Young B6129SF/J vs. Old B6129SF/*J* = 0.0980 (ns); HP-SUB: Young B6129SF/J vs. Old B6129SF/*J* = 0.0509 (ns).. TH=thalamus; FC=frontal cortex; mPFC= medial prefrontal cortex; HP-SUB=hippocampus-subiculum. T test p values: ns *p* > 0.05; ** *p* ≤ 0.01.

In order to further confirm the unusual high binding of [^18^F]nifene binding in the mPFC of the older B6129SF2/J mouse, dynamic PET/CT scan was carried out on a 16-month old B6129SF2/J mouse. Dynamic [^18^F]nifene PET/CT curves of SUV in Fig-3F of 16-month old B6129SF2/J mouse shows similar to the 11-month old mouse, a progressive increase of [^18^F]nifene uptake in all brain regions (TH, mFPC, FC, HP-SUB and CB) peaking at approximately 30 mins post-intraperitoneal injection. [^18^F]Nifene then slowly cleared from the different brain regions, except from mPFC. There was stronger retention of [^18^F]nifene in mPFC compared to the 11-month old as seen in Fig. 3F. The SUVR ratio plot for the 16-month old B6129SF2/J mouse is shown in Fig. 3G. The ratio plot for TH, FC and HP-SUB is consistent with the 11-month old, reaching a plateau after 40 min. However, mPFC was unusually higher, continued to increase due to the retention of [^18^F]nifene in mPFC, while it was clearing out from CB. At the end of the scan, SUVR for mPFC was found to be higher compared to TH (SUVR TH=1.8, mPFC=2.6, FC=1.70, HP-SUB=1.43). Thus, aging from 11-month to 16-month caused a greater amount of [^18^F]nifene to be retained in mPFC in a non-reversible manner, compared to the other brain regions. This non-reversibility of [^18^F]nifene binding in the mPFC has not been previously observed, except in our recent studies with 5xFAD mice (Liang et al., 2023). Thus, aging in B6129SF/J mice causes unusual significant retention and increase of [^18^F]nifene binding in the mPFC (*p* ≤ 0.01), while other brain regions did not exhibit significant change (Fig. 4).

### 3.3. 3xTG [^18^F]Nifene PET/CT

**Fig. 5.**
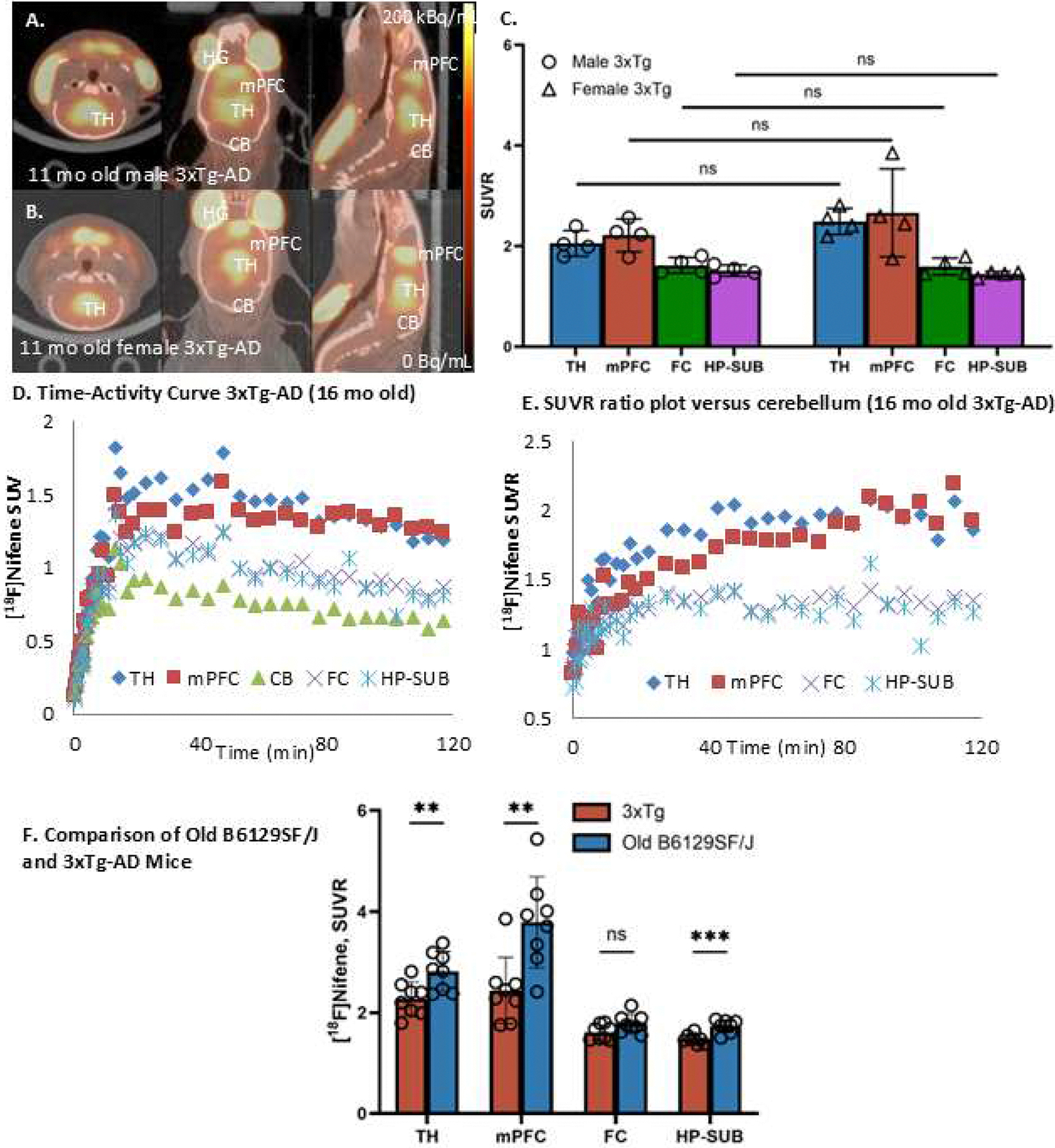
3xTg mice [^18^F]Nifene PET/CT: (A). [^18^F]Nifene in 3xTg mice (11 month male, 36*g* , 7.1 MBq, IP) showing binding in TH, FC, mPFC and low binding in CB with [^18^F]nifene greater in mPFC compared to other regions; (B). [^18^F]Nifene in 3xTg mice (11 month female, 34*g* , 7.1 MBq, IP) showing binding in TH, FC, mPFC and low binding in CB with [^18^F]nifene greater in mPFC compared to other regions; (C). Bar graph comparing SUVR averages (CB as reference) of 3xTg male (*n* = 4) and female (*n* = 4) in TH, mPFC and FC. Differences in [^18^F]nifene binding between the two groups were not significant (TH=thalamus; FC=frontal cortex; mPFC=medial prefrontal cortex; CB=cerebellum; HG=harderian gland). (D). Time activity curve of [^18^F]nifene in 16-month old 3xTg mouse (28*g* , 14.43 MBq, IP) showing uptake and clearance from various brain regions, except mPFC which showed higher uptake and slower clearance. (E). Ratio plot versus CB (SUVR) shows a ~1.86 for TH and mPFC and ~1.92 post-injection. (F). Comparison of [^18^F]Nifene PET/CT in old B6129SF/J versus 3xTg-AD mice: TH: Old B6129SF/J vs. 3xTg-AD = 0.0092 (**); mPFC: Old B6129SF/J vs. 3xTg = 0.0042 (**); FC: Old B6129SF/J vs. 3xTg-AD = 0.0552 (ns); HP-SUB: Old B6129SF/J vs. 3xTg-AD = 0.0004 (***). TH=thalamus; FC=frontal cortex; mPFC= medial prefrontal cortex; HP-SUB=hippocampus-subiculum. T test p values: ns *p* > 0.05; * *p* ≤ 0.05; ** *p* ≤ 0.01; *** *p* ≤ 0.001.

Age matched 3xTg-AD mice (11-month old from UCI Model-AD) were used in the static [^18^F]nifene PET/CT scans. The accumulation of [^18^F]nifene in the brains of the 3xTg-AD mice looked similar to the older B6129SF2/J mice (Fig. 5A,B). The highest levels of [^18^F]nifene binding was found in the mPFC, in both males and females, as opposed to the TH in the younger animals. Thus, the rank order of the binding of [^18^F]nifene changed to mPFC> TH> FC≥ HP-SUB. There was greater variability in mPFC binding of [^18^F]nifene in the 3xTg-AD mice compared to the old B6129SF2/J mice. Difference in the levels of binding between mPFC and TH was much smaller in 3xTg-AD compared to the B6129SF2/J. The SUVR values of 3xTg-AD male mice were: mPFC=2.21; TH=2.05; FC=1.62; HP-SUB=1.51 which were generally lower compared to the 3xTg-AD female mice in some regions, SUVR: mPFC=2.66; TH=2.49; FC=1.60; HP-SUB=1.44. Differences between male and female 3xTg-AD mice were not found to be statistically significant as shown in Fig. 5C.

Because of the similarity of brain distribution of 3xTg-AD and 11-month old B6129SF2/J in the PET/CT static scans (Figs. 3A,B and 5A, B), dynamic scans of 3xTg-AD mice was undertaken to compare with the time-activity curves of the B6129SF2/J mice. Dynamic [^18^F]nifene PET/CT curves of SUV in Fig-4D of 16-month old 3xTg-AD mouse shows a progressive increase of [^18^F]nifene uptake in all brain regions (TH, mFPC, FC, HP-SUB and CB) peaking at approximately 30 mins post-intraperitoneal injection. [^18^F]Nifene then slowly cleared from the different brain regions, including from mPFC. Although the uptake of [^18^F]nifene in the mPFC was high, the retention was not as strong as that observed in the 16-month old B6129SF2/J. [^18^F]Nifene clearance was similar to that observed in the TH (Fig. 5D). The SUVR ratio plot for the 16-month 3xTg-AD mouse is shown in Fig. 5E. All regions, TH, mPFC, FC and HP-SUB reached a plateau after 40 min with TH and mPFC having approximately similar ratios (SUVR TH=1.86, mPFC=1.92, FC=1.35, HP-SUB=1.26). The binding behavior of [^18^F]nifene in the mPFC region appeared to be different in the 16-month old 3xTg-AD compared to the 16-month old B6129SF2/J. There appeared to be some degree of reversibility in the binding of [^18^F]nifene in the case of the 3xTg-AD mice compared to the B6129SF2/J mice.

The 3xTg-AD mice exhibited lower [^18^F]nifene binding in the different brain regions compared to age matched old B6129SF2/J (Fig. 5F). Significant reduction in HP-SUB, TH and mPFC was seen in the 3xTg-AD when compared to old B6129SF2/J mice (reduced by >10 %), while FC was not significant. These significant differences will have to be carefully interpreted, due to the anomalous mPFC binding in the B6129SF2/J mice.

### 3.4. [^18^F]Nifene PET-MR correlation

**Fig. 6.**
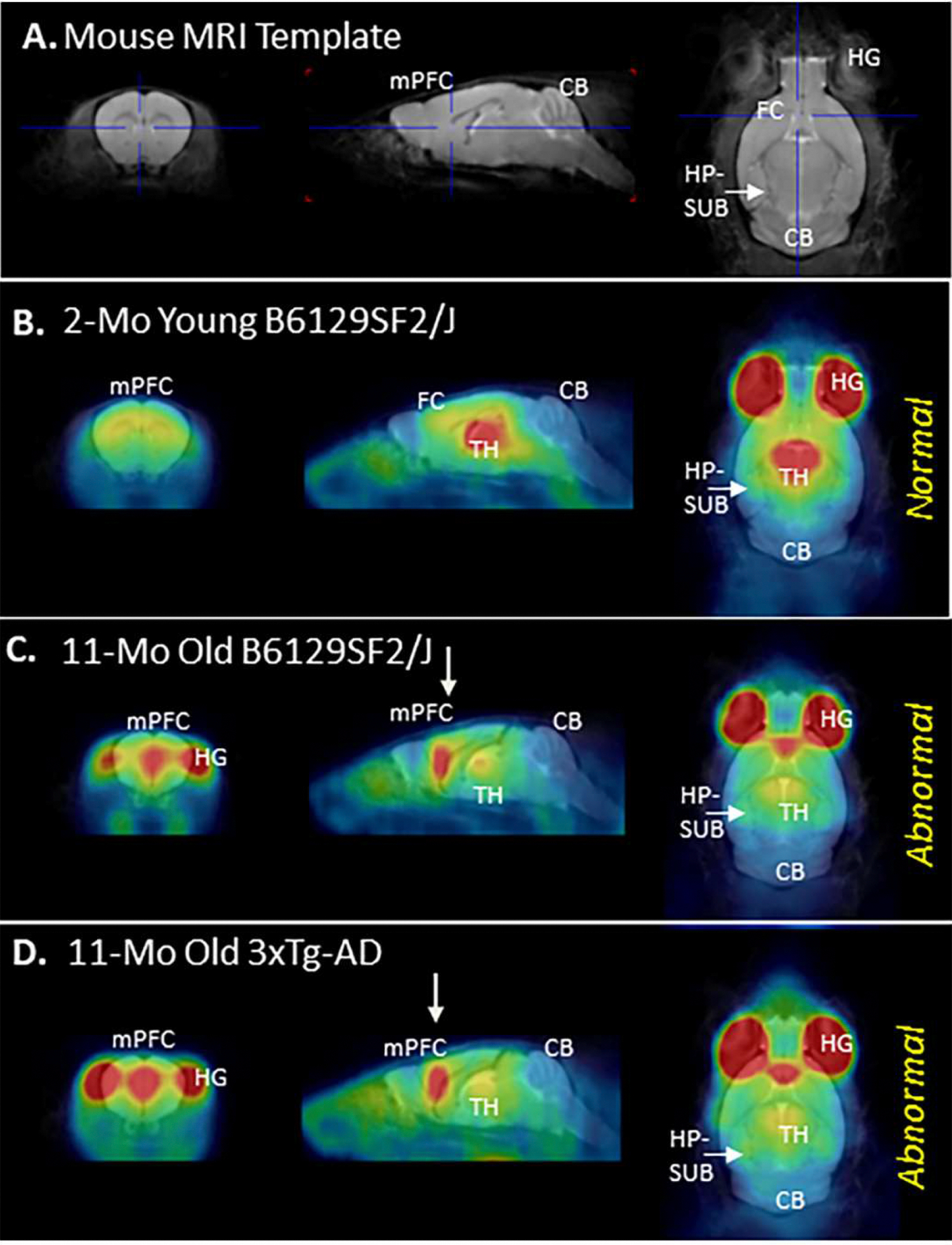
PET-MR of [^18^F]nifene 3xTG mice: (A). Coregistered [^18^F]nifene PET-MR young B6129SF2/J mouse (2 month old), coronal, sagittal and horizontal brain sections; (B). Coregistered [^18^F]nifene PET-MR mouse 3xTG, sagittal and coronal brain sections; (C-E). Three superior to inferior planes of coregistered [^18^F]nifene PET-MR 3xTG mouse with cross hairs placed on the medial prefrontal cortex/anterior cingulate in sagittal and transaxial brain sections (mPFC=medial prefrontal cortex; TH=thalamus; CB=cerebellum; HG=harderian gland; AC=anterior cingulate).

Anatomical localization of [^18^F]nifene PET with MRI co-registration was used to help further discern the potential differences in the mPFC binding in B1269SF2/J and 3xTg-AD mice (Fig. 6). Using orthogonal MRI slices from a template of normal mice (Fig. 6A) and PMOD software, PET images of the 2-month old B6129SF2/J mice (Fig. 6B), 11-month old B6129SF2/J mice (Fig. 6C) and 3xTg-AD mice (Fig. 6D) were coregistered. Bilateral regions outside of the brain corresponded to the regions around the eyes and harderian glands (HG). Within the brain, the 2-month old B6129SF2/J mice, high binding region of [^18^F]nifene corresponded to TH in the MRI followed by lower levels of binding in the FC. Cerebellum had the least levels of binding similar to other mice species (Campoy et al., 2021; Zhang et al., 2023; Liang et al., 2023). The 11-month old B6129SF2/J mice exhibited higher uptake in the mPFC region (cross-hair in the MRI template, Fig. 6A) in all the three planes (Fig. 6C). This high activity in the mPFC is localized mid-sagittally within the brain and away from HG. This higher [^18^F]nifene region aligns with reported anatomical location of mPFC (Premachandran et al., 2020; Kamigaki 2019) and ACC (van Heukelum et al., 2020) in the mouse brain. Binding in the TH was lower compared to mPFC (Fig. 5C). The 11-month old 3xTg-AD mice also exhibited the high uptake in the mPFC region, similar to 11-month old B6129SF2/J mice in all the three planes (Fig. 6D). Because of resolution issues with in vivo PET imaging, any subtle regional differences within the mPFC were not evident in Fig. 4 and 5, although PET-MRI co-registered images provided further evidence of the mid-sagittal localization of [^18^F]nifene within the brain of 3xTg-AD mice and B6129SF2/J mice.

### 3.5. Ex vivo [^18^F]Nifene

**Fig. 7.**
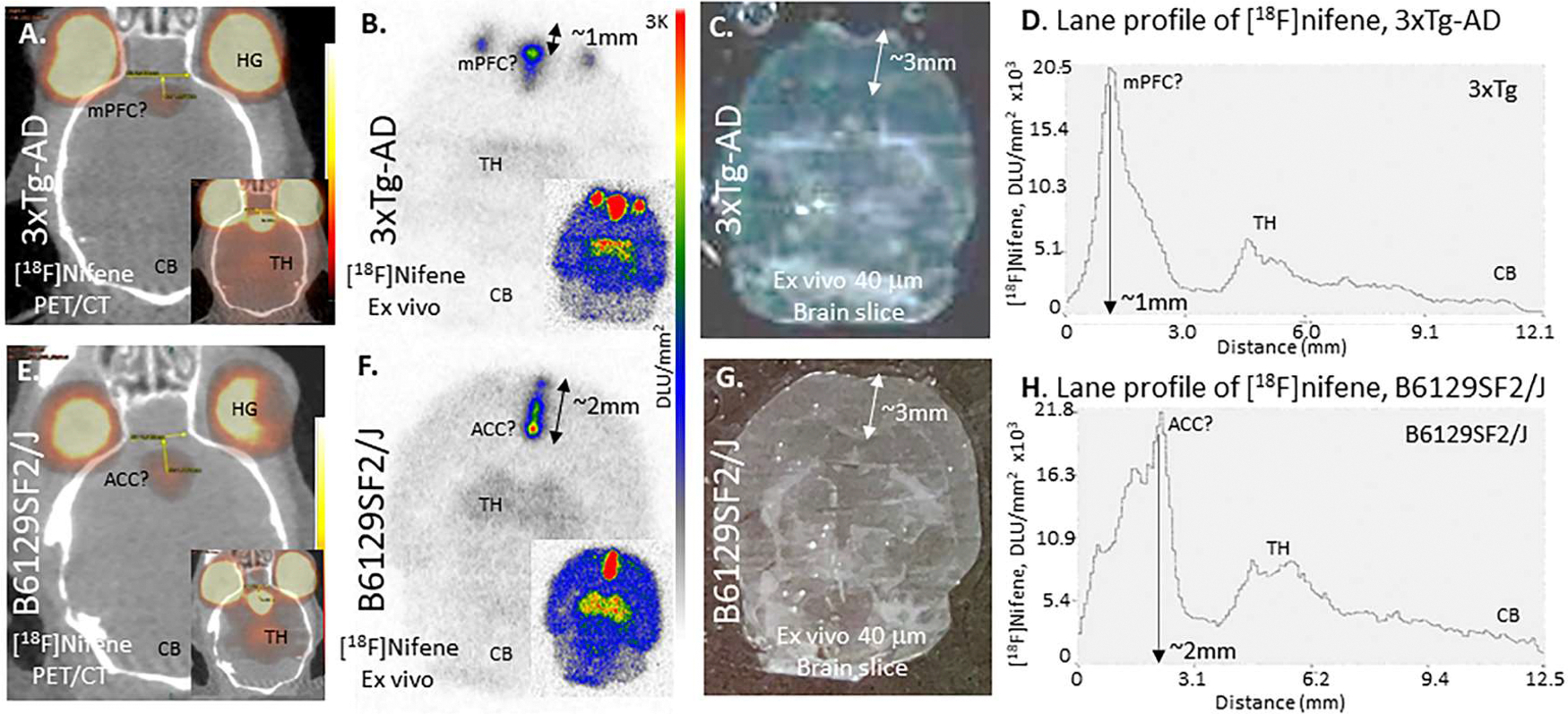
Comparison of In Vivo and Ex Vivo [^18^F]nifene mice: (A). Horizontal brain in vivo PET/CT [^18^F]nifene image in 3xTg-AD mouse brain at approx. 70 min postinjection (16-month old female 34*g* ; 7.44 MBq IP); inset shows [^18^F]nifene in TH; (B). Ex vivo [^18^F]nifene autoradiograph (40 μm thick slice) of same 3xTg-AD mouse after PET/CT experiment. [^18^F]Nifene activity in mPFC was located ~1 mm from the edge of FC; inset shows higher intensity [^18^F]nifene image in TH; (C). Scanned brain slice of 3xTg-AD mouse showing ~3 mm distance between edge of FC and corpus callosum; (D). [^18^F]Nifene lane profile from the anterior FC edge to the posterior CB edge (~12 mm). The high intensity mPFC was located at ~1 mm while the TH was ~4.5–6 mm; (E). Horizontal brain in vivo PET/CT [^18^F]nifene image in B1269SF2/J mouse brain at approx. 70 min post-injection (16-month old female 44*g* ; 14.8 MBq IP); inset shows [^18^F]nifene in TH; (F). Ex vivo [^18^F]nifene autoradiograph (40 μm thick slice) of same B1269SF2/J mouse after PET/CT experiment. High [^18^F]nifene activity in mPFC was located ~2 mm from the edge of FC, with some activity at ~1 mm; inset shows higher intensity [^18^F]nifene image in TH; (G). Scanned brain slice of B1269SF2/J mouse showing ~3 mm distance between edge of FC and corpus callosum; (H). [^18^F]Nifene lane profile from the anterior FC edge to the posterior CB edge (~12 mm). The highest intensity mPFC was located at ~2 mm, which may be ACC while the TH was ~4.5–6 mm.

Because of the continued uncertainty of the similarities or differences in the mPFC binding of [^18^F]nifene in vivo PET/CT, ex vivo autoradiography was undertaken. After the [^18^F]nifene PET/CT scan of 16-month old 3xTg-AD mice and 16-month old B1269SF2/J mice, the mice were killed and the brain excised immediately. The [^18^F]nifene brain was rapidly frozen and horizontal brain sections were obtained. Fig. 7 shows horizontal brain sections of in vivo [^18^F]nifene PET/CT and ex vivo [^18^F]nifene autoradiography of 16-month old 3xTg-AD mice and 16-month old B1269SF2/J mice.

[^18^F]Nifene activity in mPFC of 3xTg-AD was located ~1 mm from the edge of FC and appears anterior to ACC. Scanned brain slice of 3xTg-AD mouse showed corpus callosum (CC) ~3 mm posterior to edge of FC. [^18^F]Nifene lane profile from the anterior FC edge to the posterior CB edge (~12 mm) suggested the high intensity mPFC was located at ~1 mm while the TH was ~4.5–6 mm. This high intensity [^18^F]nifene localization was unlikely in the ACC. In the case of B1269SF2/J mouse brain the high [^18^F]nifene activity was located ~2 mm from the edge of FC, with some activity at ~1 mm. Lane profile suggested that [^18^F]nifene highest intensity was located at ~2 mm, which may be ACC while the TH was ~4.5–6 mm similar to 3xTg-AD. Thus, it may be surmised that the high levels of [^18^F]nifene accumulation in the midline of 3xTg-AD and B1269SF2/J mice is centered in different brain regions.

### 3.6. In vitro [^125^I]IBETA studies

**Fig. 8.**
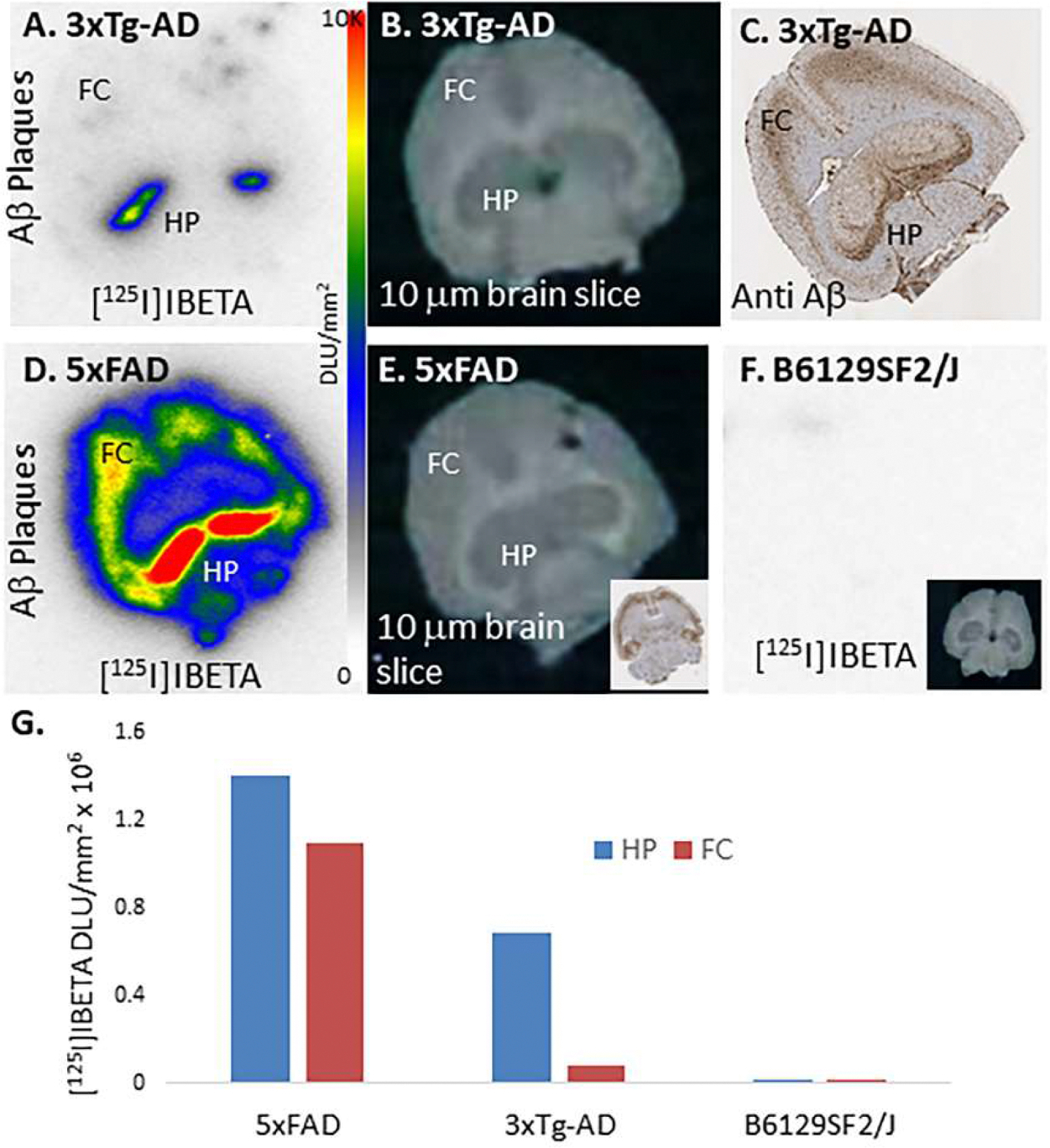
In vitro [^125^I]IBETA for Ab Plaques in 3xTG Mice: (A). [^125^I]IBETA binding to Aβ plaques in 10 μm brain slice of 3xTG transgenic mice; (B). Scan of the 3xTg brain slice showing FC and HP; (C). Adjacent slices immunostained with anti-Aβ showing location of Aβ plaques in FC and HP; (D). For comparison in the same experiment, [^125^I]IBETA binding to Aβ plaques in 10 μm brain slice of 5xFAD transgenic mice; (E). Scan of the 5xFAD brain slice showing FC and HP (inset shows anti-Aβ); (F). Absence of Aβ plaques and lack of [^125^I]IBETA binding in 10 μm brain slice of B6129SF/J mice; (G). Plot showing binding of [^125^I]IBETA in HP and FC in 5xFAD, 3xTg and B6129SF2/J mice brain slices.

In vitro binding of the Aβ plaque imaging agent, [^125^I]IBETA (Nguyen et al., 2022; Liang et al., 2023) was evaluated in brain slices obtained from 3xTg-AD mice and B6129SF2/J mice and compared with 5xFAD mice (Fig. 8). Binding of [^125^I]IBETA was seen in HP and FC in 3xTg-AD mice (Fig. 8A) and 5xFAD mice (Fig. 8D), but completely absent in the B6129SF2/J mice (Fig. 8F). Anti-Aβ immunostaining of the different brain slice also confirmed presence of Aβ plaques in the 3xTg-AD and 5xFAD mice. The higher levels of Aβ plaques in 5xFAD mice are consistent with our previous findings in 5xFAD mice (Nguyen et al., 2022; Sandhu et al., 2024). Compared to 5xFAD, level of Aβ plaques in 3xTg-AD mice were significantly lower (Fig. 8G). These findings are also consistent with lower levels of Aβ plaques in the 3xTg-AD mice model (Javonillo et al., 2022).

### 3.7. Nicotine challenge B6129SF/J mice

The irreversible binding of [^18^F]nifene in the mPFC of 16-month old B6129SF2/J compared to TH and other brain regions suggested potential differences in the binding of [^18^F]nifene to α4β2* nAChRs in the brain regions of B6129SF2/J mice. Since nicotine has been shown to compete with [^18^F]nifene in the brain in vivo very efficiently (Kant et al., 2011; Liang et al., 2023; Hillmer et al., 2011), nicotine challenge PET/CT [^18^F]nifene studies of the 16-month old B6129SF2/J mice were carried out.

**Fig. 9.**
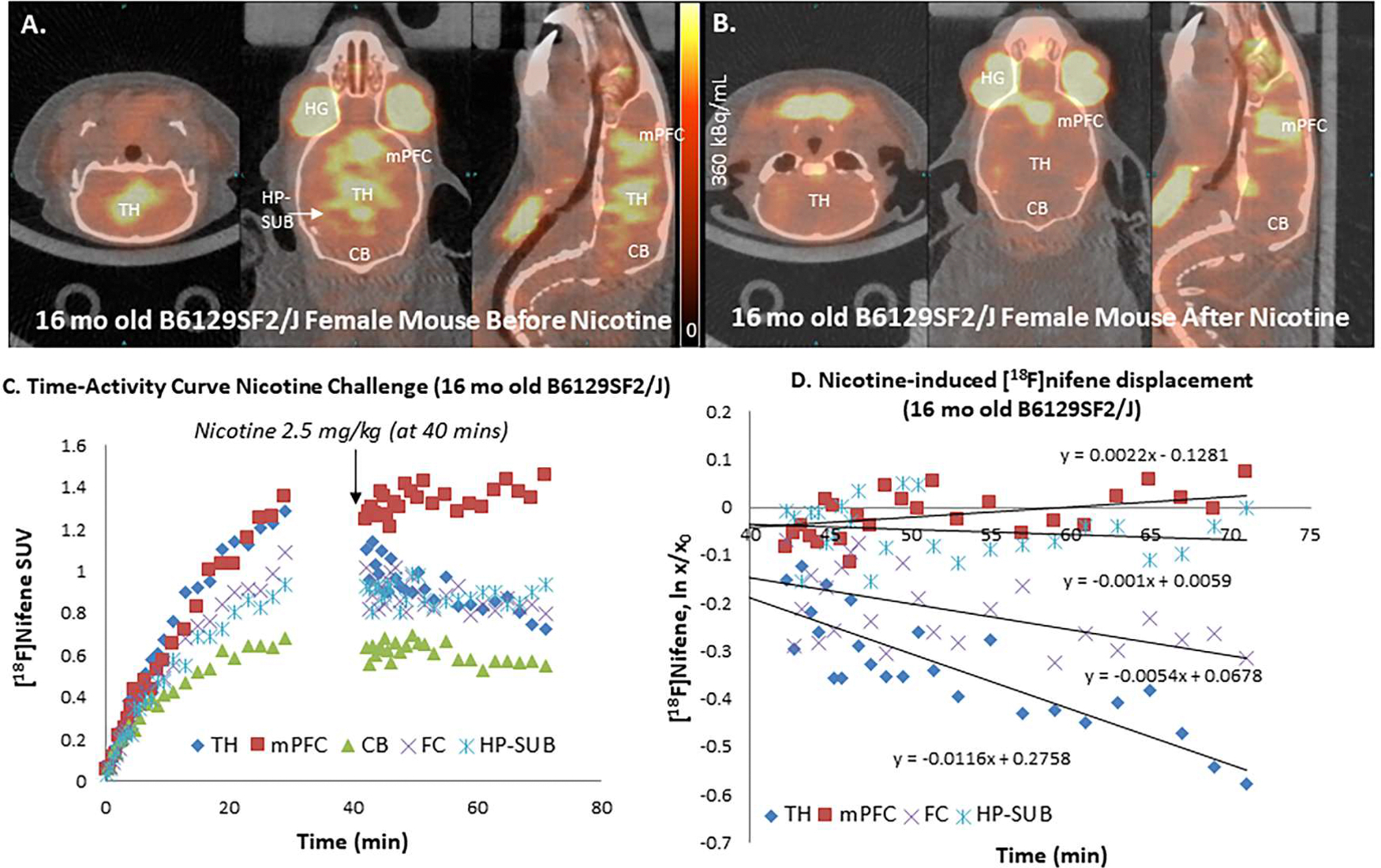
Post-Nicotine effects In Vivo: (A). PET/CT of [^18^F]nifene (10.38 MBq, IP) in control B6129SF2/J mouse female (38 g) showing coronal, transaxial and sagittal images at 30 min after nifene injection and 10 min before nicotine administration; (B). Coronal, transaxial and sagittal images at 30 min after nicotine (2.5 mg/kg, IP); (C). Time-activity curve of TH, FC, mPFC, HP-SUB and CB showing nicotine intervention (arrow); (D). Dissociation rate (ln X/X_0_) plot for TH= k_off_ =0.012 min^−1^, FC= k_off_ =0.005 min^−1^ and HP-SUB= k_off_ =0.001 min^−1^. Association rate in mPFC was found to be 0.002 min^−1^. (TH=thalamus; FC=frontal cortex; mPFC= medial prefrontal cortex; HP-SUB=hippocampus-subiculum; CB=cerebellum).

Fig. 9A shows orthogonal PET/CT brain slices of [^18^F]nifene in the 16-month old B6129SF2/J mouse prior to injection of nicotine. Expected [^18^F]nifene binding was seen in the TH and mPFC regions including FC. Subsequent to nicotine administration (IP, 2.5 mg/kg, 40 min postinjection of [^18^F]nifene), [^18^F]nifene binding was displaced from TH, but [^18^F]nifene was not displaced from mPFC as seen in all the 3-planes in Fig. 9B. Time-activity curves in Fig. 9C show similar increasing uptake of [^18^F]nifene in the TH and mPFC while FC and HP-SUB exhibited similar rising levels at lower levels. Cerebellum plateaued within 30 mins post-injection. Nicotine induced a rapid displacement of [^18^F]nifene from TH, FC and HP-SUB but there was no displacement or change in the [^18^F]nifene bound to mPFC (Fig. 9C). Measured off-rates greater for TH (koff =0.012 min^−1^), FC (koff =0.005 min^−1^), HP-SUB (koff =0.001 min^−1^) (Fig. 9D). The displaceability of [^18^F]nifene from TH and FC of normal mice is consistent with our recent findings (Liang et al., 2023; Zhang et al., 2023). The measured nicotine-induced TH off-rate of [^18^F]nifene was similar to that previously reported for the rat TH (koff = 0.06 min^−1^; Kant et al., 2011).

### 3.8. Nicotine challenge 3xTg-AD mice

The time-activity uptake curves of B6129SF2/J and 3xTg-AD mice were distinctly different (Figs. 3 and 4). Additionally, because of the small regional differences in the mPFC binding of [^18^F]nifene between the 16-month old B6129SF2/J and 3xTg-AD and the irreversibility of nicotine-induced [^18^F]nifene in the mPFC of 16-month old B6129SF2/J, effect of nicotine in the 3xTg-AD was warranted. Using similar protocol as described above, nicotine challenge in the 3xTg-AD mouse was carried out.

**Fig. 10.**
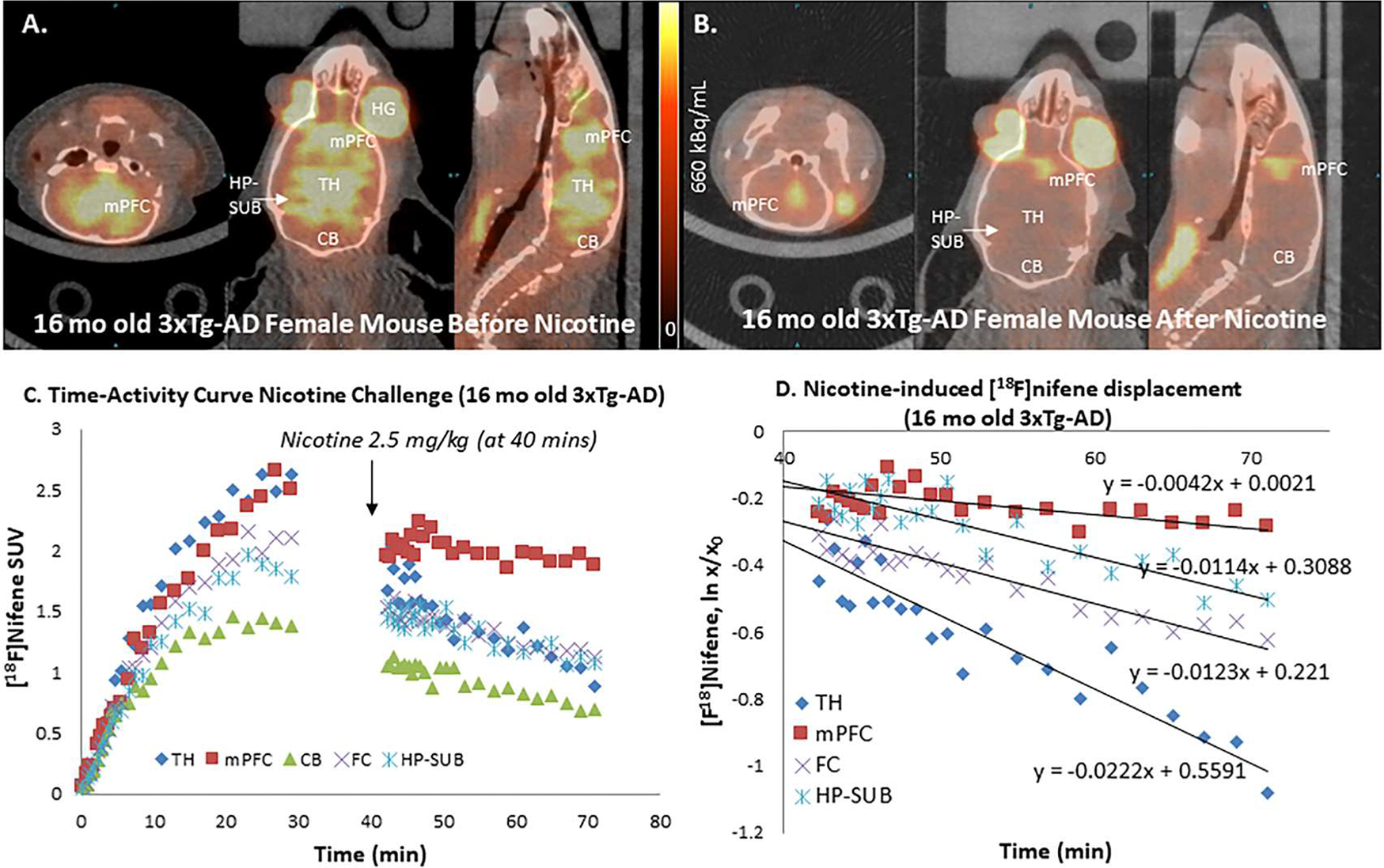
Post-Nicotine effects In Vivo: (A). PET/CT of [^18^F]nifene (10.99 MBq, IP) in control B6129SF2/J mouse female (38 g) showing coronal, transaxial and sagittal images at 30 min after nifene injection and 10 min before nicotine administration; (B). Coronal, transaxial and sagittal images at 30 min after nicotine (2.5 mg/kg, IP); (C). Time-activity curve of TH, FC, mPFC, HP-SUB and CB showing nicotine intervention (arrow); (D). Dissociation rate (ln X/X_0_) plot for TH= k_off_ =0.022 min^−1^, FC= k_off_ =0.012 min^−1^, HP-SUB= k_off_ =0.011 min^−1^ and mPFC= k_off_ =0.004 min^−1^. (TH=thalamus; FC=frontal cortex; mPFC= medial prefrontal cortex; HP-SUB= hippocampus-subiculum; CB=cerebellum).

Fig. 10A shows orthogonal PET/CT brain slices of [^18^F]nifene in the 16-month old 3xTg-AD mouse prior to injection of nicotine. Similar high [^18^F]nifene binding was seen in the TH and mPFC regions followed by FC, HP-SUB and least in the CB. Subsequent to nicotine administration (IP, 1 mg/kg, 40 min postinjection of [^18^F]nifene), [^18^F]nifene binding was displaced from TH, and there appeared to be partial [^18^F]nifene displacement from mPFC as seen in all the 3-planes in Fig. 10B. Time-activity curves in Fig. 10C show similar increasing uptake of [^18^F]nifene in the TH and mPFC while FC and HP-SUB exhibited similar rising levels at lower levels. Cerebellum plateaued within 30 mins post-injection. Nicotine induced a rapid displacement of [^18^F]nifene from TH, FC and HP-SUB and there was slower displacement of [^18^F]nifene bound to mPFC (Fig. 10C). Measured off-rates greater for TH (koff =0.022 min^−1^), FC (koff =0.012 min^−1^), HP-SUB (koff =0.01 min^−1^) and mPFC (koff =0.004 min^−1^) (Fig. 10D). Compared to B6129SF2/J mice which showed no displacement of [^18^F]nifene from mPFC, the binding of [^18^F]nifene in the mPFC region of 3xTg-AD mouse was partially displaceable. The measured nicotine-induced TH off-rates of [^18^F]nifene was similar to that previously reported for the 5xFAD TH (Liang et al., 2023).

### 3.9. Pretreatment with nicotine

In order to evaluate if the α4β2* nAChR binding sites in the B6129SF2/J mice and 3xTg-AD mice were saturable, pre-injection of nicotine studies were carried out. Using our previously reported studies, nicotine was injected IP (1 mg/kg, free base, injected as the ditartarate salt) 5 min prior to [^18^F]nifene for static PET/CT studies. Using this protocol and nicotine dose, our previous studies have shown that binding of [^18^F]nifene is suppressed in all brain regions, suggesting saturation of α4β2* nAChR binding sites by nicotine. Both 16 month old B6129SF2/J and 3xTg-AD mice were investigated.

**Fig. 11.**
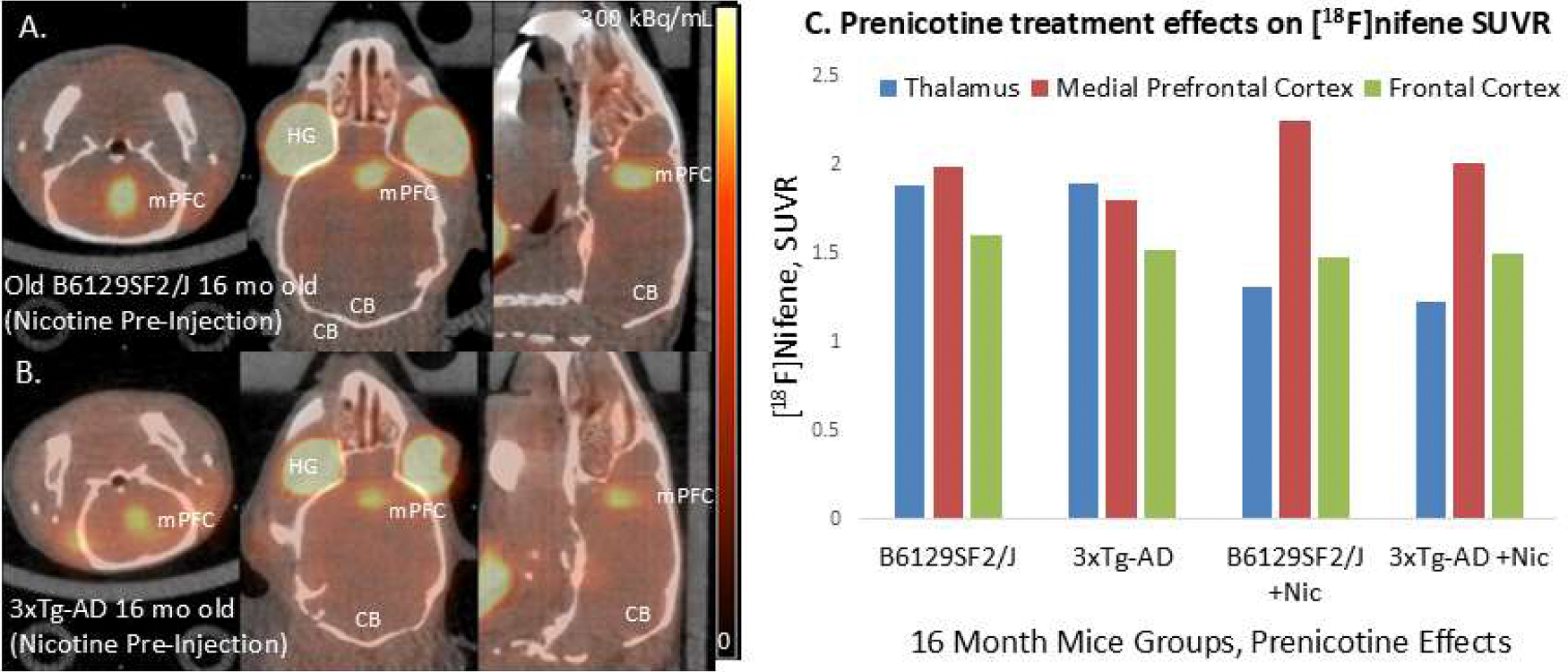
PreNicotine effects In Vivo: (A). PET/CT of [^18^F]nifene (10.73 MBq, IP) in control B6129SF2/J mouse male (24 g) showing coronal, transaxial and sagittal images after nicotine preadministration; (B). PET/CT of [^18^F]nifene (9.58 MBq, IP) 3xTg-AD mouse female (34 g) showing coronal, transaxial and sagittal images after nicotine preadministration; (C). Plot showing [18F]nifene binding in the absence and presence of prenicotine in B6129SF2/J mice and 3xTg-AD mice (B6129SF2/J SUVR: TH=1.89; mPFC=1.99; FC=1.60; 3xTg-AD SUVR: TH=1.89; mPFC=1.81; FC=1.52; B6129SF2/*J*+Nic SUVR: TH=1.31; mPFC=2.25; FC=1.48; 3xTg-AD+Nic SUVR: TH=1.23; mPFC=2.01; FC=1.50). (TH=thalamus; FC=frontal cortex; mPFC= medial prefrontal cortex; CB=cerebellum).

Effect of pre-saturation of α4β2* nAChR binding sites with nicotine in the 16-month old B6129SF2/J mouse is seen in Fig. 11A. Orthogonal PET/CT brain slices of [^18^F]nifene show blocking of the receptors in the TH and other brain regions. However, mPFC binding of [^18^F]nifene is still present, suggesting that this site is non-saturable and [^18^F]nifene binding is irreversible and non-displaceable. In the case of the 16-month old 3xTg-AD mouse, pre-saturation with nicotine successfully blocked TH and FC similar to the B6129SF2/J mouse, however mPFC was still present (Fig. 11B,C). Upon closer examination of the SUVR plot (Fig. 11C), after prenicotine, there was a 11 % greater reduction in [^18^F]nifene bound to the mPFC in the 3xTg-AD mouse compared to B6129SF2/J mouse (B6129SF2/J SUVR: TH=1.31; mPFC=2.25; FC=1.48; 3xTg-AD SUVR: TH=1.23; mPFC=2.01; FC=1.50). This suggests and supports the earlier ex vivo and uptake kinetics that there appears to be a difference in the mPFC brain region between the B6129SF2/J and 3xTg-AD mice, with some potential overlap.

## 4. Discussion

We previously investigated the potential effects of Aβ plaques in the 5xFAD mice on the binding of [^18^F]nifene to α4β2* nAChRs (Liang et al., 2023). A significant finding was the high localized mPFC [^18^F]nifene binding in the 5xFAD mice, a region which did not directly correlate with a high [^125^I]IBETA binding to Aβ plaques. Additionally, this binding of [^18^F]nifene in the mPFC region displayed poor reversibility compared to other brain regions. The presence of abundant microglia in the 5xFAD mice is well known (Crapser et al., 2020; Forner et al., 2021). Since mPFC is innervated with nicotinic receptors (Koukoouli et al., 2016), altered or dysfunctional nicotinic receptors along with the increased levels of microglia in the mPFC could potentially adversely affect cholinergic functions in the 5xFAD mice resulting in impaired cognitive processes.

In this work we examined [^18^F]nifene binding in 3xTg-AD mice which have been reported to express both Aβ plaques and Tau pathologies prominently by 12-months (Javonillo et al., 2022). The study was undertaken to assess if this AD mice model would have similar mPFC anomalous binding of [^18^F]nifene like the 5xFAD model. For comparison, B6129SF2/J mice were used as the control animals. The binding of [^18^F]nifene in the B6129SF2/J mice was highly unusual presenting the highest [^18^F]nifene accumulation in the mPFC region in all the older male and female animals. In younger 2-month old B6129SF2/J animals this anomalous binding was not observed. This [^18^F]nifene uptake in the mPFC region was higher than the levels seen in the TH, binding was irreversible and resistant to displacement by nicotine, unlike what was observed in the TH and in younger animals. Since microglia markers in B6129SF2/J animals do not vary significantly from ages 4 to 18 months (Javonillo et al., 2022), this aging effect causing the unusual high uptake of [^18^F]nifene may be due to other factors. In our numerous studies of [^18^F]nifene PET/CT imaging in different species of mice, including C57BL/6 (Liang et al., 2023; Zhang et al., 2023) and BALB/c mice (Constantinescu et al., 2013), β2 knock-out mice (Bieszczad et al., 2012; Zhang et al., 2023), A53T α-synucleinopathy transgenic mice (Campoy et al., 2021), such anomalous mPFC binding has not been observed. The B6129SF2/J mice were fully characterized to be absent of any Aβ plaques and Tau. The 2-month old B6129SF2/J mice at 11-month of age began to exhibit similar abnormal mPFC binding of [^18^F]nifene. Therefore, it appears that it is an age-related phenomenon in these control B6129SF2/J which has not been observed in any of the other wild-type mice species with [^18^F]nifene PET studies.

Age related (11-month old) 3xTg-AD mice also exhibited similar unusual [^18^F]nifene binding in the mPFC region in males and females. However, there were subtle differences between 3xTg-AD mice and the 11-month old B6129SF2/J mice. Although the binding of [^18^F]nifene in the mPFC region was high and comparable to that observed in the TH, it was not completely irreversible as in the case of the B6129SF2/J mice. Treatment with nicotine was able to displace small amount of [^18^F]nifene from the mFPC, unlike the B6129SF2/J mice which was resistant to the effects of nicotine. Further detailed analysis of [^18^F]nifene bound to the B6129SF2/J and 3xTg-AD mouse brain was carried out by using co-registration of [^18^F]nifene PET with mouse brain MR template. Thalamic and extrathalamic regions were confirmed using the PET-MR co-registered images, including the extracranial localization in the vicinity of the harderian glands in both B6129SF2/J and 3xTg-AD mice. Evaluation across the different planes suggested very focused [^18^F]nifene localization in the ACC and mPFC, brain regions critical for executive control (Kamigaki, 2019; Premachandran et al., 2020; Van Heukelum et al., 2020). However, it was difficult to identify subregional differences in localization of [^18^F]nifene within the mPFC in the B6129SF2/J and 3xTg-AD mice.

**Fig. 12.**
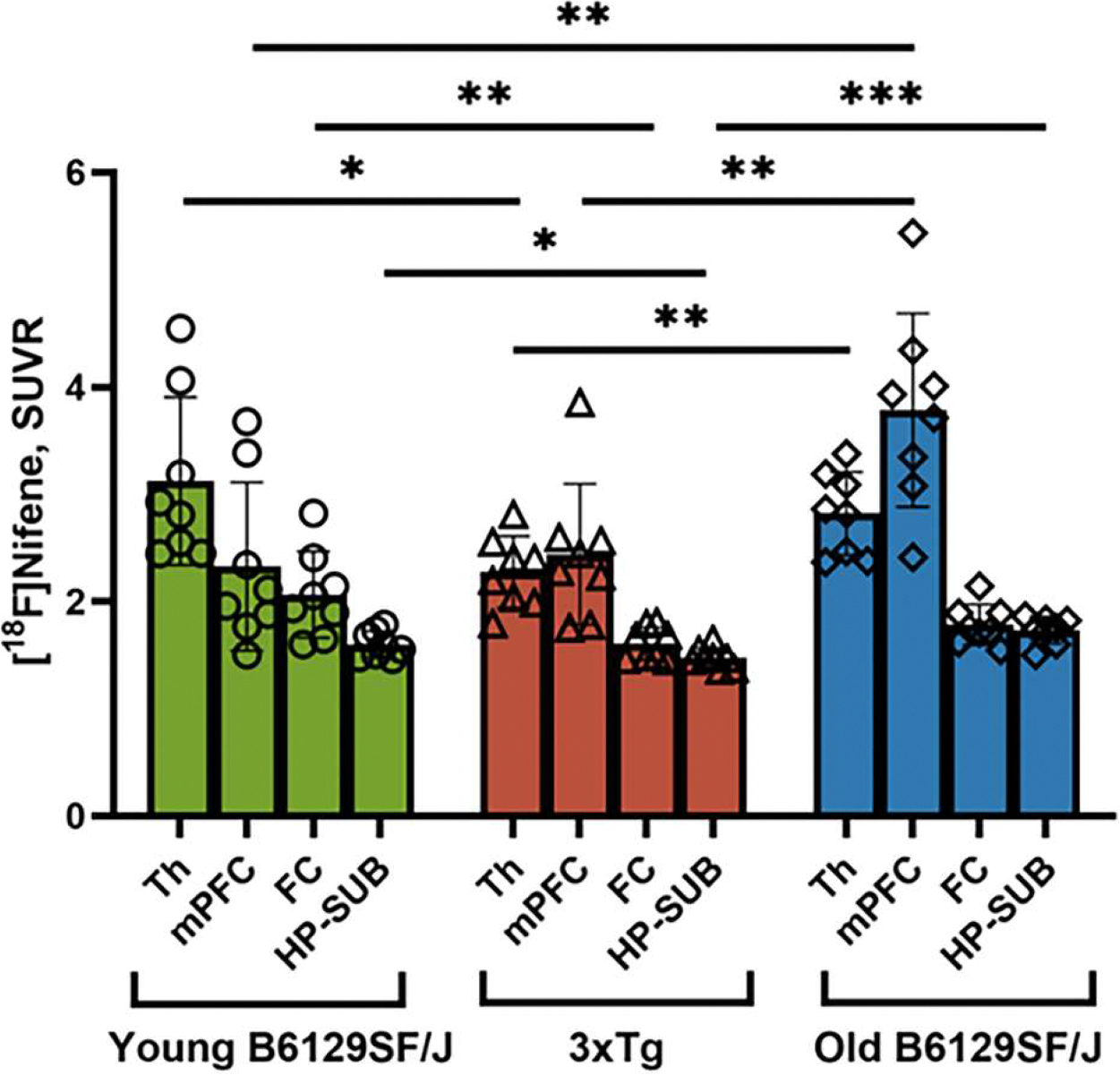
Comparison of [^18^F]Nifene PET/CT in the 3 groups: TH: Young B6129SF/J vs. 3xTg-AD = 0.0136 (*); Young B6129SF/J vs. Old B6129SF/*J* = 0.3428 (ns); Old B6129SF/J vs. 3xTg-AD = 0.0092 (**); mPFC: Young B6129SF/J vs. 3xTg-AD = 0.7641 (ns); Young B6129SF/J vs. Old B6129SF/*J* = 0.0039 (**); Old B6129SF/J vs. 3xTg = 0.0042 (**); FC: Young B6129SF/J vs. 3xTg-AD = 0.0094 (**); Young B6129SF/J vs. Old B6129SF/*J* = 0.0980 (ns); Old B6129SF/J vs. 3xTg-AD = 0.0552 (ns); HP-SUB: Young B6129SF/J vs. 3xTg-AD = 0.0401 (*); Young B6129SF/J vs. Old B6129SF/*J* = 0.0509 (ns); Old B6129SF/J vs. 3xTg-AD = 0.0004 (***). TH=thalamus; FC=frontal cortex; mPFC= medial prefrontal cortex; HP-SUB=hippocampus-subiculum. T test p values: ns *p* > 0.05; * *p* ≤ 0.05; ** *p* ≤ 0.01; *** *p* ≤ 0.001.

Comparison of the three groups of mice is shown in Fig. 12.The TH of 3xTg-AD mice had lower [^18^F]nifene binding (reduced byapproximately 20 %) compared to both young B6129SF2/J and was significant. The mPFC [^18^F]nifene binding was significantly higher in the old B6129SF2/J compared to both the young B6129SF2/J and the 3xTg-AD mice (>150 %). Frontal cortex was reduced in the 3xTg-AD compared to the young B6129SF2/J (reduced by >20 %). Significant reduction in HP-SUB was seen in the 3xTg-AD when compared to both the young and old B6129SF2/J mice (reduced by >10 %). Overall, the 3xTg-AD mice exhibited lower [^18^F]nifene binding in the different brain regions compared to age matched old B6129SF2/J.

To further delineate the mPFC binding of [^18^F]nifene in the B6129SF2/J and 3xTg-AD mice, ex vivo studies were carried out after the PET/CT scan. Ex vivo autoradiography of the 3xTg-AD brain after the in vivo PET/CT scan confirmed the high [^18^F]nifene binding in the mPFC (Fig. 7B), consistent with what was observed in the in vivo PET/CT (Fig. 7A). Similarly, ex vivo autoradiography of the B6129SF2/J brain after the in vivo PET/CT scan confirmed the high [^18^F]nifene binding in the mPFC (Fig. 7F), consistent with what was observed in the in vivo PET/CT (Fig. 7E). However, the location of the primary focus of [^18^F]nifene binding in the two brains were different. The B6129SF2/J focus was more closer to the ACC region, while 3xTg-AD was anterior to ACC by approximately a millimeter. Exact anatomical location of these regions will have to be carefully examined. Both these areas are mid-sagittal and very focused. There also appears to be a certain amount of overlap in the binding as seen in the lane plot in Figs. 7D,H.

The abnormally high uptake, irreversibility and non-saturable binding of [^18^F]nifene in the mPFC remains unresolved in the case of both transgenic species 5xFAD and 3xTg-AD. The additional uncertainty introduced as a result of the present work is the similar unusual binding in the B6129SF2/J mice. This unusual binding of [^18^F]nifene is unlikely due to potential microbleeds (Koveri et al., 2015; Shih et al., 2018; Cacciottolo et al., 2021). Our results with sodium [^18^F]fluoride to assess any blood brain barrier (BBB) disruption did not show any brain uptake in the 5xFAD mice. Brain metastasis with BBB disruption have been imaged using sodium [^18^F]fluoride (Gori et al., 2015). Similarly, our results with [^18^F]FDG and other reported findings of [^18^F]FDG in the 5xFAD and 3xTg-AD mice have not reported any unusual FC increases (Nicholson et al., 2010; Franke et al., 2020), thus potentially ruling out BBB disruption. Overexpression of α4β2* receptors were observed in microglia/macrophages and astrocytes due to ischemia induced in rats (Martin et al., 2015).

The reduction of [^18^F]nifene binding in the HP-SUB of 3xTg-AD mice is consistent with our observations of a reduction in HP-SUB of postmortem human AD (Karim et al., 2025). A reduction in cholinergic nerve terminals has been reported to occur even before neuritic plaques in transgenic AD mice (German et al., 2003). However, interpretation of the results in the FC from the transgenic AD mice are confounded due to the unusual mPFC binding. Our findings also suggest that the molecular nature of in vivo [^18^F]nifene binding in the mPFC of 3xTg-AD mice is different compared to the B6129SF2/J mice. It is unclear if this unusual mPFC binding of [^18^F]nifene in the older B6129SF2/J is manifested in the similar unusual binding in the 3xTg-AD mice. Since PFC is innervated with nicotinic receptors (Koukoouli et al., 2016), these receptors are altered or dysfunctional in the mPFC and adversely affect cholinergic functions in the 3xTg-AD mice resulting in impaired cognitive processes. However, because of the unusual binding of [^18^F]nifene in B6129SF2/J mice in the mPFC, interpretation of anomalies in the 3xTg-AD mice in this brain region is not possible. It should be noted that the B6129SF2/J mice have been used as wild-type in a number of studies as controls for various cognitive tasks.

## 5. Conclusion

The 3xTg-AD transgenic mice had reduced [^18^F]nifene binding compared to B6129SF2/J controls, suggesting possible effects of Aβ plaques, Tau and microglia on α4β2* nAChRs. The anomalous higher, non-displaceable binding of [^18^F]nifene in the mPFC confounded accurate measurements in the FC. Similar, high binding of [^18^F]nifene in the mPFC region was observed in the 5xFAD mice. Limitations of the study include several unanswered questions: Does the increased [^18^F]nifene binding in mPFC in the 5xFAD and 3xTg-AD mice suggest a compensatory mechanism against Aβ and microglia pathology? The irreversibility and inability of nicotine to displace in vivo [^18^F]nifene raises several possibilities: (1). An interaction of Aβ oligomers, fibrils, plaques and microglia with the α4β2* receptor that alters pharmacological properties of the receptor and affects the binding of [^18^F]nifene and nicotine; (2). The ability of [^18^F]nifene to bind and an inability of nicotine to displace bound [^18^F]nifene in the B6129SF2/J mice suggests additional interactions of [^18^F]nifene at the receptor site or an alternate site, such as the microglia, rendering it irreversible. Additional studies are needed to address this anomalous behavior of [^18^F]nifene.

## Figures and Tables

**Table 1:** Details of mice used in the in vivo nifene PET/CT imaging study.

Mice Type	Age, month	Sex	Weight	[^18^F]Nifene^4^ MBq, ip	Aβ swe	PS1 EP^6^	Source

Young B6129SF2/J^1^	2 11-13	Male *N* = 4	20-26g 52-64g	4.2 - 6.7 8.7 - 10.1	WT	WT	JAX
Young B6129SF2/J^1^	2 11-13^7^	Female *N* = 4	14-20g 32-48g	4.96 - 6.1 8.6 - 10^7^	WT	WT	JAX
Old B6129SF2/J^2^	11-16	Male *N* = 4	30-36g	5.92-6.66 13 (TAC)^7^	WT^5^	WT^6^	MODEL-AD UCI
Old B6129SF2/J^2^	11-16	Female *N* = 4	32-40g	5.33-5.92 13.1-14.8 (for static scan)	WT^5^	WT^6^	MODEL-AD UCI
3xTg-AD^3^	11-16	Male *N* = 4	28-36g	6-7.77 14.4 (TAC)	HO^5^	HO^6^	MODEL-AD UCI
3xTg-AD^3^	11-16	Female *N* = 4	30-38g	6.7-7.6 9.6, 11^7^	HO^5^	HO^6^	MODEL-AD UCI

1 Young B61229SF/J mice were purchased from Jackson Labs

2 Old B61229SF/J mice were obtained from UCI MODEL-AD project

3 Age and sex matched old 3xTg-AD mice were obtained from UCI MODEL-AD project.

4 [^18^F]Nifene in saline injected intraperitoneally (ip) in all animals.

5 Mice were characterized for presence (homozygous; HO) or absence (wild type; WT) of the Aβ swe mutation.

6 Mice were characterized for presence (HO) or absence (WT) of presenilin1 (PS1).

7 Nicotine challenge experiments.

## Data Availability

Data will be made available on request.
